# Temporal Dynamics of Depressive Symptoms and Cognitive Decline in the Oldest Old: Dynamic Time Warp analysis of the Leiden 85-plus Study

**DOI:** 10.1192/j.eurpsy.2024.189

**Published:** 2024-08-27

**Authors:** A. J. van der Slot, A. S. Bertens, S. Trompet, S. P. Mooijaart, J. Gussekloo, F. van den Bos, E. J. Giltay

**Affiliations:** ^1^Department of Psychiatry, Leiden University Medical Center; ^2^Mental Health Service Rivierduinen; ^3^Department of Internal Medicine, Section of Gerontology and Geriatrics; ^4^Department of Public Health and Primary Care, Leiden University Medical Center, Leiden; ^5^Health Campus, Leiden University, The Hague, Netherlands

## Abstract

**Introduction:**

The prevalence of depressive symptoms and cognitive decline increases with age, reducing quality of life. However, the temporal relationship between the two remains elusive.

**Objectives:**

We aimed to explore the temporal relationship between depressive symptoms and cognitive decline in individuals aged 85 years, during up to 5 years follow-up.

**Methods:**

Participants eligible for this study were selected from the Leiden 85-plus Study, who participated for at least 3 follow-up measurements. Depressive symptoms were assessed at baseline and at follow-up in a period of 6 yearly assessments, utilizing the 15-item Geriatric Depression Scale (GDS-15). Cognitive decline was measured through various tests including the Mini Mental State Exam (MMSE), Stroop Test, Letter Digit Coding Test, and immediate and delayed recall using the 12-word learning test. Dynamic Time Warping (DTW) analysis was employed to model their temporal dynamics, in undirected and directed analysis, to ascertain whether depressive symptoms precede cognitive decline, or vice versa.

**Results:**

The study included a total of 325 (54.2%) of 599 patients, of whom 68.0% were female, 45.0% with intermediate to higher education, and all aged 85 years. Depressive symptoms and cognitive functioning significantly covaried in time, and directed analyses showed that depressive symptoms preceded most of the parameters of cognitive decline in the oldest old. Of the 15 GDS symptoms, those with the strongest outstrength were worthlessness, hopelessness, low happiness, dropping activities/interests, and low satisfaction with life (all p<.01).

**Conclusions:**

We found a strong temporal link between depressive symptoms and subsequent cognitive decline in a population of the oldest old. This highlights the importance of a holistic approach that considers both mental and cognitive well-being in the aging population. As depressive symptoms were an early indicator of cognitive decline, it is of importance that healthcare professionals recognize and address depressive symptoms early to allow for appropriate interventions and support, to potentially mitigate the impact on cognitive decline.

**Disclosure of Interest:**

None Declared

